# Advancement Perforator Cheek Flap for Aesthetic One-Stage Reconstruction of Postoncological Extended Split-Thickness Defects of the Nasal Sidewall

**DOI:** 10.1155/2013/169208

**Published:** 2013-10-27

**Authors:** Matteo Rossi, Arianna Milia, Marco Carmisciano, Salvatore D'Arpa, Adriana Cordova, Francesco Moschella

**Affiliations:** Plastic and Reconstructive Surgery, Department of Surgical, Oncological and Stomatological Sciences, University of Palermo, Via del Vespro 129, 90127 Palermo, Italy

## Abstract

Aesthetic reconstruction of soft tissue nasal sidewall loss has an important influence on the appearance of the nose. The unique character of this subunit and the complex relationships with a number of different facial or nasal subunits make the excision of large tumors difficult to manage. Numerous techniques are described in the literature, but a primary reconstruction with a final good result is not often possible. The authors develop an advancement cheek flap for an aesthetic one-stage reconstruction of postoncological extended nasal sidewall defects. Between January 2009 and July 2012, 16 patients (mean age, 63.3 yr) underwent excision of skin tumors of nasal sidewall and immediate reconstruction with an advancement cheek flap nourished by perforators from the transverse facial branch of the superficial temporal artery. The tumors were excised with 0.4–0.6 cm lateral margins and defects size ranged from 2.6 × 2.6 cm to 3.5 × 5 cm. Oncological radicality was obtained in all cases. The aesthetic results were excellent in all patients. No scar revision was needed. The authors' advancement cheek flap can be considered the first choice for reconstruction of split-thickness defect of nasal sidewall larger than 2.5 cm because it reestablishes in one stage the nasal contour detail.

## 1. Introduction

 Thirty percent of nonmelanoma skin cancers occur on the nose, of which sixteen percent is located on the sidewall [1]. The nasal sidewall is a combination of convex and concave elements extending laterally from the dorsum to the junction of the nose with the cheek. The skin is thin in the rhynion area and becomes thicker caudally where it is separated from the ala by the alar groove. Various aesthetic facial and nasal subunits (eyebrow, lower eyelid, cheek, nasal ala, and nasal dorsum) are contiguous in this area, and the contours of the tissues change so evidently between them that the excision of large tumors is very difficult to manage.

 Numerous techniques, such as a full thickness skin graft, V-Y flaps, nasolabial flaps, paramedian forehead flap, and supratrochlear artery perforator propeller flap (STAPP flap) are described in the literature, but a primary aesthetic reconstruction is not often possible [1–5]. Commonly, nasal sidewall defect larger than 2 cm requires a two-stage procedure or if it occupies an adjacent facial subunits, it is preferable to reconstruct each area in a modular fashion, addressing the portion of the defect in each aesthetic unit separately [1, 3–5]. The natural translation of this concept is “multiple flaps with multiple scars.”

 To select a valid reconstructive technique, it is helpful to assess the mobility and laxity of skin surrounding the defect and determine which facial structures may be distorted by secondary tissue movement. Particularly important in this regard are nasal ala, lip, lower eyelid, and eyebrow.

 The medial cheek area could be considered an ideal donor site. The skin is thicker and more mobile than the other units of the face. The fibrous attachment of the superficial muscular aponeurotic system (SMAS) in the melolabial crease, relaxed skin tension lines located in the lower eyelid, and the boundary line at the inferior orbital rim provide important landmarks for concealing incision [6].

 Classically, cheek flaps are used for cheek, lower eyelid, and temporofrontal region reconstruction [7–9]. In nose reconstruction they are most often employed to restore small nasal sidewall defects up to 2.5 cm in size or in combination with the paramedian forehead flap or glabellar flap if the defects involve the infraorbital unit [2–4, 10, 11].

 In this paper the authors describe the design, execution, and results of an advancement cheek flap for an aesthetic single-stage reconstruction of postoncological extended nasal sidewall defects larger than 2.5 cm.

## 2. Materials and Methods

 Between January 2009 and July 2012 the authors' technique was performed in sixteen patients to reconstruct split-thickness defects of nasal sidewall after malignant tumors resection. The authors received approval by the Department Review Board according to local Institutional Review Board Standards and also obtained the informed consent from the patients before the study. 

 There were nine male and seven female patients with age ranging between 54 and 74 years (mean, 63.31 yr). On histology, eleven patients had basal cell carcinomas (superficial, nodular, ulcerate, multifocal, and sclerodermiforme type) and five had spinal cell carcinomas. The lesions were located only on the nasal sidewall in seven cases and in nine cases included also adjacent nasal or facial subunits. The tumors were excised with 0.4–0.6 cm lateral margins and the defects size ranged between 2.6 × 2.6 cm and 3.5 × 5 cm (median, 3.0 × 3.35 cm). All patients underwent immediate reconstruction using an advancement cheek flap according to the technique described below (Table 1).

### 2.1. Surgical Technique

 The advancement cheek flap proposed is a pedicle laterally based flap. The major vascular supply is derived from the transverse facial branch of the superficial temporal artery [7].

Preoperative pinch test is necessary to evaluate medial cheek movement. If the pinched skin is smaller than the expected defect size, the flap cannot be harvested.

 Under local anesthesia, excision of the tumor is obtained to establish oncological radicality and the flap is marked. The first incision passes from the inferior aspect of the defect and is outlined in the nasofacial sulcus and melolabial crease; the second incision passes from the superior aspect of the defect to the lateral canthus and it can be placed in a subciliar line or along the inferior bony orbital rim. Placing the superior incision in the subciliar line results in a less conspicuous scar and avoids prolonged lower eyelid edema but requires the removal of normal eyelid skin and includes a risk for ectropion especially in the elderly patients. 

**Figure 1:**
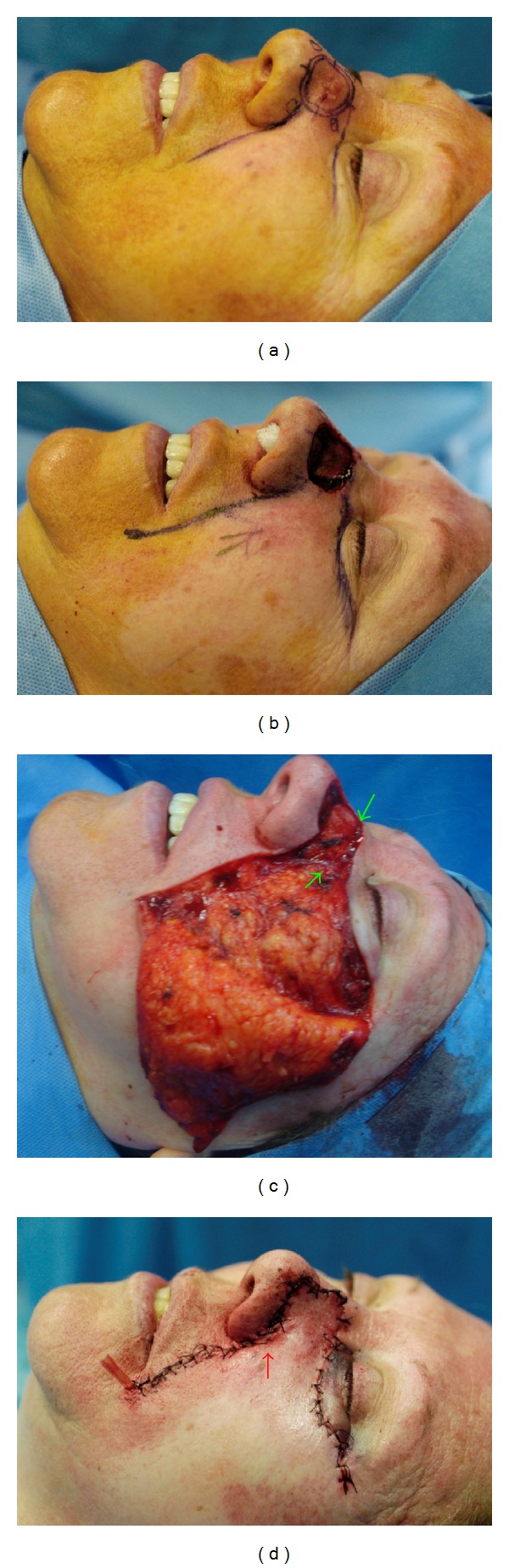
Patient no. 2 showed in Table 1. (a) Basal cell carcinoma of the left nasal sidewall and dorsum marked for the excision; (b) 2.6 × 2.8 cm defect after tumor resection. Advancement cheek flap designed with inferior incision outlined in the nasofacial sulcus and melolabial crease and superior incision placed in the inferior bony orbital rim. (c) Flap elevated from the medial border in a subcutaneous plane. Green arrows indicate the placement of the two adsorbable anchor sutures. (d) Intraoperative final result. Red arrow indicates the Burow's triangle excised from the inferomedial aspect of the flap to recreate alar-facial sulcus.

Flap dissection proceeds from the medial to the lateral border in a supra-SMAS plane, preserving orbicularis oculi muscle, buccal branch of the facial nerve, and malar fat pad to avoid facial deformity. Care is taken not to damage perforating vessels from the transverse facial branch of the superficial temporal artery that lie laterally in the cheek. Only the perforating vessels that limit the flap's movement are sacrificed. This ensures vitality to the distal portion of the flap and allows to reduce intraoperative risk of bleeding and postoperative hematomas. The flap is advanced to the defect without any tension and anchored with two absorbable (polyglactin 3/0) sutures to the maxillary and nasal bone periosteum to avoid loss of nasofacial sulcus and lower eyelid retraction. Excess of subcutaneous tissue can be removed from the medial border of the flap to match the thickness of the nasal defect. Standing cutaneous deformities created by flap's movement are excised superiorly at the junction line between the cheek and the lower eyelid and inferiorly in the melolabial crease. A Burrow's equalizing triangle is resected from the inferior-medial aspect of the flap to recreate the alar groove. The skin flap is sutured with a few polyglactin 5/0 stitches and then with a simple interrupted 5/0 nylon suture. A Penrose drain is inserted for 24 h (Figure 1).

## 3. Results

**Figure 2:**
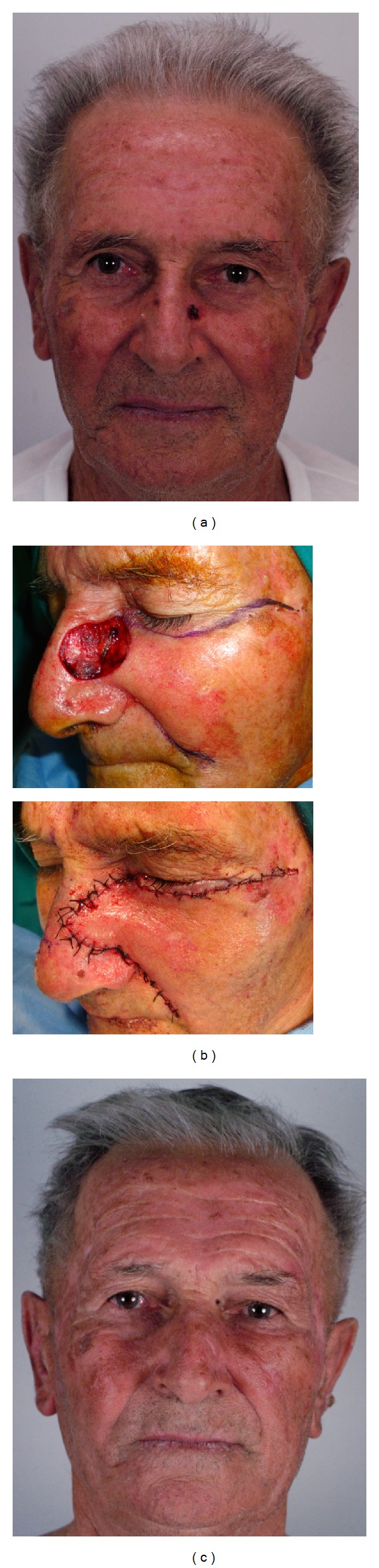
Patient no. 8 showed in Table 1. (a) Basal cell carcinoma of the left nasal sidewall and infraorbital unit; (b) (intraoperative view) 3 × 3.2 cm defect after tumor resection. Advancement cheek flap is marked (up). Flap inset (down). (c) Final result 6 months after operation.

**Figure 3:**
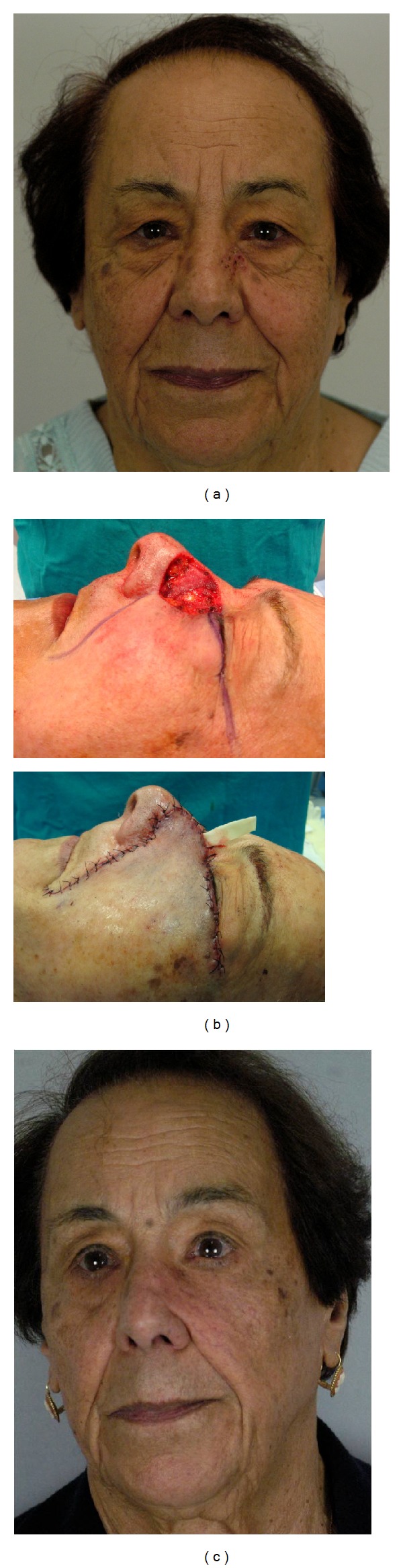
Patient no. 12 showed in Table 1. (a) Basal cell carcinoma of the left nasal sidewall and infraorbital unit; (b) (intraoperative view) 3.2 × 3.8 cm defect after tumor resection. Advancement cheek flap designed with inferior incision outlined in the nasofacial sulcus and melolabial crease and superior incision placed in a subciliar line (up). Flap inset (down). (c) Final result 24 months after operation.

**Figure 4:**
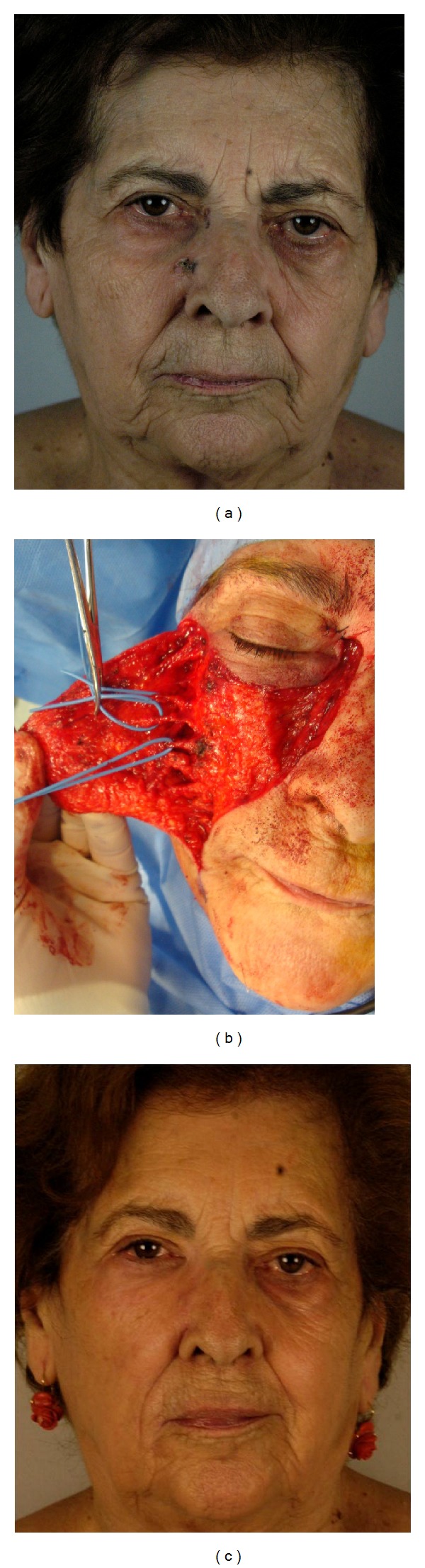
Patient no. 13 showed in Table 1. (a) Multifocal basal cell carcinoma of the right nasal sidewall, medial canthal, and infraorbital unit; (b) (intraoperative view) 3.5 × 3.8 cm defect after tumor resection. Flap elevation in a subcutaneous plane. Identification and preservation of the perforating vessels from the transverse facial branch of the superficial temporal artery. (c) Final result 24 months after operation.

 Oncological radicality was obtained. There was no tumor recurrence during the follow-up period (range, 3 months to 36 months). The aesthetic results were excellent in all patients (Figures 2, 3, and 4). There was no partial or total flap loss. One case of temporary lower eyelid edema was observed when an inferior bone orbital rim incision was chosen. No scar revision was needed.

## 4. Discussion

 An aesthetic single-stage reconstruction of large split-thickness defects on the nasal sidewall is still one of the most difficult aims to achieve.

 Moolenburgh et al. [1] proposed an algorithm of treatment for nasal sidewall only with skin defects larger than 1.5 cm. They recommended the use of full thickness skin graft, nasolabial flaps, and paramedian forehead flap.

Full-thickness skin grafts, although allowing a single stage reconstruction, have a typical “patch” appearance caused by color mismatch and contour defects [2, 12].

 In the authors' experience, the V-Y flap is usually aesthetically superior to full-thickness skin grafts, but the pincushion effect is very common and often requires a second procedure to recreate the nasofacial sulcus [13, 14].

 A disadvantage of all nasolabial flaps in males is the transfer of hair-bearing skin to the nose and generally the tendency to a “trap door” appearance. Distortion of the melolabial crease can occur for defects larger than 2.5 cm [2, 3, 15].

Paramedian forehead flap and supratrochlear artery perforator propeller flap undeniably achieve aesthetically good results, but they are indicated when more than two nasal subunits are involved [4, 5].

 A common problem of all these procedures is that if the nasal sidewall defect encompasses adjacent facial subunit, it is preferable to treat each area separately so that more than one flap or graft is needed to avoid disruption of important aesthetic landmarks such as the alar groove, nasofacial sulcus, or melolabial crease.

 Advancement cheek flap reestablishes in one stage the nasal contour detail respecting the facial anatomy. It is created by convergent incisions that allow a sliding movement of adjacent cheek tissue in a single vector towards the defect and ensure a better lymphatic drainage. The skin of the medial aspect of the flap is more mobile than the other units of the face and normally is not covered by a beard pattern in men [6]. Scars are best camouflaged because the incisions are placed in relaxed skin tension lines or in borders between aesthetic regions of the face. Identification and preservation of the perforator vessels originated from the transverse facial branch of the superficial temporal artery guarantee a good vascularization to the distal portion of the flap and reduce the risk of hematomas. Anchoring the flap to the maxillary bone and nasal bone periosteum avoids lower eyelid retraction. The possibility to remove the fat excess from the medial aspect of the flap ensures a perfect conformation to the defect avoiding loss of the nasofacial sulcus. The excision of a Burrow's triangle from the inferior-medial aspect of the flap allows to recreate the alar groove.

## 5. Conclusion

 The authors' advancement cheek flap can be considered the first-choice technique that allows an aesthetic reconstruction of split-thickness defects of the nasal sidewall in a single stage and with a single donor site without distorting surrounding functional and aesthetic structures. It is indicated for defects between 2.6 × 2.6 cm up to 3.5 × 5 cm in size and extended to nasal dorsum, medial canthal, and infraorbital units. It is most applicable to older patients with skin excess and who will heal with better scars.

## Figures and Tables

**Table 1:** Series of patients treated with advancement cheek flap.

Pat. no.	Age	Sex Female/male	Subunit	Tumor type	Margins resection (cm)	Defect size *H* (cm) × *W* (cm)	Complications	Followup (Months)
1	54	F	Right nasal sidewall	Superficial basal cell carcinoma	0.4	2.6 × 2.6	—	6
2	54	F	Left nasal sidewall + nasal dorsum	Nodular basal cell carcinoma	0.4	2.6 × 2.8	—	12
3	58	F	Right nasal sidewall	Superficial basal cell carcinoma	0.4	2.8 × 2.8	—	9
4	62	M	Right nasal sidewall	Spinal cell carcinoma	0.6	3.0 × 2.6	—	24
5	59	M	Left nasal sidewall	Superficial basal cell carcinoma	0.4	2.8 × 3.0	—	16
6	68	M	Left nasal sidewall	Spinal cell carcinoma	0.6	3.0 × 2.8	—	18
7	58	M	Right nasal sidewall	Spinal cell carcinoma	0.6	3.2 × 3.0	—	27
8	72	M	Left nasal sidewall + left infraorbital	Ulcerate basal cell carcinoma	0.4	3.0 × 3.2	—	6
9	66	M	Right nasal sidewall	Spinal cell carcinoma	0.6	3.0 × 3.5	—	30
10	59	F	Left nasal sidewall + nasal dorsum + left infraorbital	Spinal cell carcinoma	0.6	3.0 × 3.8	—	12
11	61	M	Right nasal sidewall + nasal dorsum + right infraorbital	Superficial basal cell carcinoma	0.4	3.2 × 3.6	—	22
12	62	F	Left nasal sidewall + left infraorbital	Superficial basal cell carcinoma	0.4	3.2 × 3.8	—	24
13	74	F	Right nasal sidewall + right medial canthal + right infraorbital	Multifocal basal cell carcinoma	0.6	3.5 × 3.8	Temporary right lower eyelid edema	24
14	70	M	Right nasal sidewall + nasal dorsum + right infraorbital	Ulcerate basal cell carcinoma	0.4	3.5 × 4	—	12
15	65	F	Right nasal sidewall + right infraorbital	Ulcerate basal cell carcinoma	0.4	3.5 × 4.2	—	21
16	71	M	Left nasal sidewall + left infraorbital + left medial canthal	Sclerodermiform basal cell carcinoma	0.6	3.5 × 5	—	3
